# What is the role of self-esteem in adolescents with idiopathic scoliosis under a conservative treatment?

**DOI:** 10.1186/1748-7161-8-S1-O60

**Published:** 2013-06-03

**Authors:** E D'Agata, C Pérez-Testor, S Negrini, M Rigo

**Affiliations:** 1 Fund. Hosp. Univers. Vall D’Hebron. Institut de recerca. Barcelona, Spain; 2 Research Group of Couple and Family. Ramon LLull University, Barcelona, Spain; 3 University of Brescia, Brescia, Italy; 4 IRCCS Don Gnocchi, Milan, Italy; 5 Institut Elena Salvá, Barcelona, Spain

## Background

Self-esteem is defined as a “positive or negative orientation toward oneself” or “a feeling of self-appreciation” (Rosenberg, 1965). In AIS literature, there are contrasting results about the relation between Self-esteem and treatments (Masso et al., 2002; Zhang et al, 2011).

## Aim

Evaluating any correlations between Self-esteem with Health Related Quality of Life (HRQOL) and Body Satisfaction in adolescents with IS.

## Methods

36 Italian and Spanish adolescents with IS (aged 13.1 years; Mean Cobb angle 29°; Risser 0) were visited for the first time in two different centers (Milan and Barcelona). 14 of them were treated by brace and physiotherapy, 22 only by physiotherapy. Patients answered to Rosenberg self- esteem test, SRS-22, and to Body Satisfaction questionnaires, at the beginning and six months later. Each group was divided into two subgroups, according to the self -esteem scoring: lower values (21-30) and higher values (31-40). Group with low self-esteem was composed of 14 patients, while the other by 22 patients.

## Results

Self-esteem correlated with Body Satisfaction and HRQOL (all its factors) significantly (p<0.01). Group with high Self Esteem worsened Function (p=0.003) but improved Body Satisfaction (p=0.01) in Wilcoxon text. Group of low Self Esteem (79% treated by physiotherapy) improved Self Image.

## Discussions

Self-esteem correlates with HRQ0L and Body Satisfaction, so that a positive experience of one’s own body, and QoL, are associated with higher levels of self-esteem.

Furthermore, Body Satisfaction could be considered a more general construct than Self Image, which belongs to SRS-22 questionnaire. So, people with higher Self-esteem are able to generalize the results of the conservative treatment to all their body, while patients with lower self-esteem, mainly treated by physiotherapy, focused more on Self Image related to their spine and could not appreciate the benefits to the entire body. Future research will aim at increasing sample size for a better understanding of the behaviour of the group of Low self Esteem treated by brace.

## References

[B1] Cahill S Mussap AJ Emotional reactions following exposure to idealized bodies predict unhealthy body change attitudes and behaviors in women and men J Psychosom Res 2007 62 6 631 639 10.1016/j.jpsychores.2006.11.001 17540220

[B2] Payne WK 3rd Ogilvie JW Resnick MD Kane RL Transfeldt EE Blum RW Does scoliosis have a psychological impact and does gender make a difference? Spine 1997 22 12 1380 1384 10.1097/00007632-199706150-00017 9201842

[B3] Tones M Moss N Polly DW Jr. A review of quality of life and psychosocial issues in scoliosis Spine (Phila Pa 1976) 2006 31 26 3027 3038 10.1097/01.brs.0000249555.87601.fc 17173000

